# No association between gain in body mass index across the life course and midlife cognitive function and cognitive reserve—The 1946 British birth cohort study

**DOI:** 10.1016/j.jalz.2011.09.228

**Published:** 2012-11

**Authors:** Emiliano Albanese, Rebecca Hardy, Andrew Wills, Diana Kuh, Jack Guralnik, Marcus Richards

**Affiliations:** a MRC Unit for Lifelong Health and Ageing, Department of Epidemiology and Public Health, Royal Free and University College Medical School, London, United Kingdom; b Laboratory of Epidemiology, Demography and Biometry, National Institute on Aging, Bethesda, MD, USA

**Keywords:** Epidemiology, Cognitive function, Body size, Adiposity, Vascular risk factors

## Abstract

**Background:**

The association between lifelong body mass index (BMI) and cognitive function has not been comprehensively studied.

**Methods:**

In more than 2000 men and women born in 1946, we tested associations between BMI gain at 15, 20, 26, 36, 43, and 53 years with respect to the previous measure (gain at age 15 years with respect to BMI at age 11 years), and semantic fluency (animal naming) and cognitive reserve (the National Adult Reading Test) at age 53 years, and verbal memory (word list recall) and speed/concentration (letter cancellation) at ages 43 and 53 years. Measures of BMI gain were adjusted in stages for childhood intelligence, education, socioeconomic position (SEP), lifestyle, and vascular risk factors.

**Results:**

Independent of childhood intelligence, BMI gain between ages 26 and 36 years was associated with lower memory scores (β per SD increase in BMI in men = −0.11; 95% confidence interval [CI]: −0.19, −0.02), verbal fluency (β in women = −0.11; 95% CI: −0.20, −0.02), and lower National Adult Reading Test score (β in women = −0.08; 95% CI: −0.15, −0.01), but not with speed/concentration (β in men = 0.02; 95% CI: −0.11, 0.07). Associations were largely explained by educational attainment and SEP (*P* ≥ .10). However, BMI gain at 53 years in men was independently associated with better memory (β = 0.12; 95% CI: 0.03, 0.22), and both underweight (β = −1.54; 95% CI: −2.52, −0.57) and obese (β = −0.30; 95% CI: −2.52, −0.57) women at 53 years had significantly lower memory scores.

**Conclusion:**

The adverse effect of higher BMI gain on midlife cognitive function and cognitive reserve is independent of childhood intelligence but not of education and SEP. The independent association between greater BMI gain in midlife and better cognitive function deserves further investigation.

## 1 Introduction

The graying of societies worldwide will be responsible for a steep rise in dementia cases in the coming years, and primary prevention is of great importance [1]. The identification of modifiable risk factors for dementia onset and severity, including vascular [2], metabolic [3], and nutritional [4] factors, has received increasing attention in the past decade. High adiposity is a global epidemic [5], and its link with dementia is biologically plausible [6] and of great public health importance; however, the nature of the association is complex and ambiguous [7]. On one hand, lower body size and weight loss can precede the onset of the disease by decades [8], [9], probably as a biomarker of dementia pathology itself [10]. On the other hand, higher adiposity in early midlife (i.e., before 40 years) has been shown to be associated with dementia [11], [12], lower cognitive function [13], and steeper cognitive decline in late midlife (≥50 years) [14]. It is possible, however, that the direction of this association changes with age in the same individuals, which a longitudinal approach based on multiple measures of body mass index (BMI) across the life course may help clarify. The 1946 British birth cohort study provides a unique opportunity to investigate this in a large, prospective, population-based study with repeated measures of height and weight across adulthood, cognitive function tested in late midlife, and a wide range of potential confounders. Our aim was to test associations between BMI change at multiple ages from adolescence through midlife, and midlife cognitive function and cognitive decline, adjusting for childhood intelligence, educational attainment, socioeconomic position (SEP), mental health, and a range of cardiovascular risk factors. We hypothesized that BMI gain in early adulthood is independently associated with faster cognitive decline in late midlife and with lower general cognitive ability at the same age.

## 2 Methods

### 2.1 Participants

The Medical Research Council (MRC) National Survey of Health and Development (NSHD) is a socially stratified cohort of 5362 newborns sampled from all the single legitimate births that occurred during 1 week in March 1946 in England, Scotland, and Wales. Extensive information on sociodemographic circumstances, health, and cognitive function has been obtained in adolescence and regularly thereafter [15]. In adulthood, data were collected during home visits by trained research nurses. In 1989 and 1999, data were collected on 3262 and 3035 study members, respectively, who were, in most respects, representative of the U.K. population of a similar age [16]. The North Thames Multicentre Research Ethics Committee and local boards approved the study, and informed consent was obtained from all participants.

### 2.2 Procedures

**Fig. 1:**
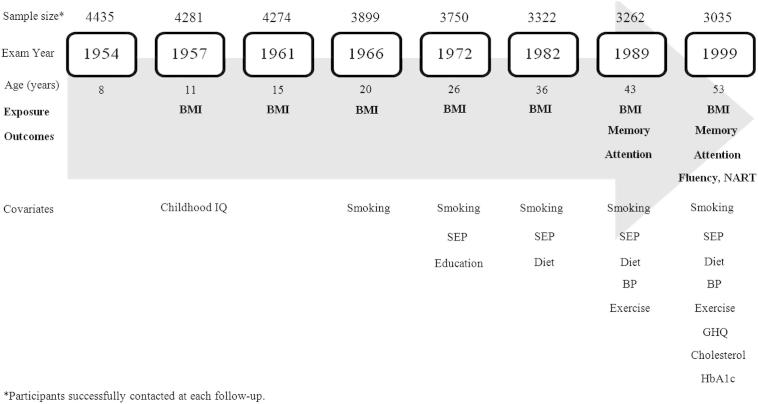
The National Survey of Health and Development. Examination years and relative study members’ age in years illustrate when outcome, exposure, and covariate measures relevant to the present study have been assessed. Abbreviations: BMI, body mass index; NART, National Adult Reading Test; SEP, socioeconomic position; BP, blood pressure; GHQ, General Health Questionnaire; HbA1C, glycated hemoglobin (%).

Years and corresponding ages at each NSHD follow-up are illustrated in Fig. 1.

The outcome in the present study was cognitive function assessed in midlife. Verbal memory was assessed at 43 and 53 years by a three-trial 15-item word list learning task (maximum score = 45) devised by the study. Verbal fluency at 53 years was measured as the total number of different animals named in 1 minute. General verbal ability (cognitive reserve) was assessed at 53 years using the National Adult Reading Test (NART) [17], with the score inverted so that higher values (maximum = 50) represented better performance. Attention, speed, and concentration were assessed using a visual letter search task at 43 and 53 years [18].At ages 11, 15, 36, 43, and 53 years, weight (in kilograms) and standing height (in centimeters) were measured according to standard protocol, and BMI (weight [kg]/height [m]^2^) was calculated and also categorized according to World Health Organization criteria (BMI of <18.5 = underweight; 18.5–24.9 = normal weight; 25–29.9 = overweight; ≥30 = obese). Height and weight were self-reported at 20 and 26 years.

Childhood cognitive ability, which was treated as a potential confounder in this study, was measured when participants were 8 years old. Their teachers or a trained carer assessed their verbal and nonverbal ability by administering the following tests: (1) 60-item nonverbal picture logic and intelligence; (2) reading comprehension, consisting of 35 sentences to be completed by choosing an appropriate word; (3) pronunciation of 50 words; and (4) vocabulary based on the same 50 words. An overall cognitive score was derived summing the standardized individual test results. The 35 standard items of the Watts Vernon Reading Test (sentence completion) was used at age 26 years to assess verbal ability, with an additional 10 items of increased difficulty to avoid a ceiling effect (maximum score = 45) [19].

Consistent with previous studies [20], [21] on adiposity and cognitive function, we considered the following health, lifestyle, and SEP confounders:

• Educational attainment by age 26 years in five categories ranging from no education to higher qualifications (degree or higher).
• Social class in six categories based on main occupation between ages 26 and 43 years according to the Registrar General system [22].
• Self-reported mental health at age 53 years using the General Health Questionnaire-28 with a standard 4-point scoring system (higher scores reflecting more severe conditions) [23].
• Physical activity (none, 1–4 times a month, or more than 4 times a month) defined as taking part in any sports, exercises, or vigorous leisure activities in the month preceding the interview at both 43 and 53 years.
• Lifetime smoking derived by combining questions asked at each age from 20 to 53 years, and defined as follows: 1, never smoked; 2, predominantly nonsmoker (a nonsmoker for at least three data collections); 3, predominantly smoker (a smoker at four or more of the data collections; 4, lifelong smoker (a smoker at all available data collections).
• At the ages of 36, 43, and 53 years, participants completed food diaries over 5 days. Data were processed by a program based on McCance and Widdowson’s *The Composition of Foods and Supplements* [24] to obtain mean percentage of kilocalories from fat consumed at 36, 43, and 53 years.
• Systolic blood pressure and diastolic blood pressure were measured at age 43 years using a Hawksley random zero sphygmomanometer, and at age 53 years by an Omron HEM-705 (Omron Corp., Tokyo, Japan) automated digital oscillometric sphygmomanometer. The second blood pressure reading was used for analysis, except in cases when only the first reading was available.
• Total cholesterol (mmol/L), and glycated hemoglobin (%) levels were assayed from a nonfasting venous blood sample at 53 years [25].

### 2.3 Statistical analysis

On inspection, all outcome measures were approximately normally distributed. We carried out descriptive statistics of raw measures by gender and then standardized all measures to obtain a mean equivalent to 0 and an SD equivalent to 1 to allow direct comparisons. We used linear regression analysis to assess the associations between BMI, separately at each age from 15 to 53 years, and each cognitive test score at age 43 and age 53 years, testing any departures from linearity of the association by addition of a quadratic term. Stages of covariate adjustment are shown in Table 1. Because the NSHD data were collected at fixed ages, we were able to test the effect of BMI change between consecutive follow-ups by adjusting each BMI measure for the BMI measure at the previous age (model 1). In a second set of models, we adjusted for cognitive ability at age 8 years (model 2). In three further sets of models, we added potential confounders (entered as continuous or categorical variables, as appropriate, see previous text) to model 2 in three stages. We adjusted for educational level and lifelong social class (model 3); smoking, physical activity level, general health, percent of kilocalories from fats (model 4); systolic and diastolic blood pressure at age 43 and 53 years and total cholesterol level and glycated hemoglobin level at age 53 years (model 5). To assess the association between BMI and cognitive decline between 43 and 53 years, we reran the aforementioned models as conditional models of change in cognitive function by adjusting standardized memory and search speed scores at age 53 years for their corresponding scores at age 43 years. Finally, to test the hypothesis that lower BMI gain over the life course is associated with higher cognitive reserve in midlife (53 years), we used the NART score at age 53 years conditioned on the Watts–Vernon score at age 26 years. At each age, the sample size for analysis was based on available BMI data at a given age in those with complete data for the specified cognitive outcome and complete covariate data at that age (sample sizes at each age are provided in Fig. 1 and in Supplementary Tables 1-3). Analyses were conducted separately for men and women, after significant gender × BMI interactions. All analyses were performed using STATA, 10th edition (STATA Corp., College Station, TX) [26].

## 3 Results

### 3.1 Study sample characteristics

**Fig. 2:**
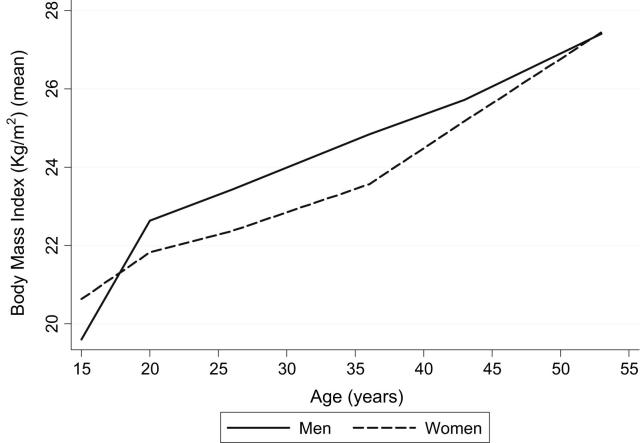
Mean BMI of men and women in the National Survey of Health and Development by age.

The sample sizes for those interviewed at each age are reported in Fig. 1. Overall, in the restricted sample of those with complete data for all cognitive measures in midlife (n = 2195), 67.3% also had complete data for BMI measures at all ages. The characteristics of the restricted sample are shown in Table 2 by gender. Mean BMI at all ages did not differ between those with and without complete midlife cognitive measures (all *P* values >.4). Those with complete BMI information at all ages (n = 1607) had similar scores for memory at 43 years (*P* = .34) and for letter cancellation accuracy at 43 years (*P* = .16) and 53 years (*P* = .66), but higher scores for memory (*P* = .044), NART (*P* = .066), and verbal fluency (*P* = .001) at 53 years and had a higher mean childhood cognitive score (*P* < .001). BMI (kg/m^2^) increased through adulthood, such that by age 53 years, mean BMI exceeded the standard overweight threshold of 25 kg/m^2^ in both men (27.4) and women (27.5) (Fig. 2).

### 3.2 BMI and midlife cognitive function

**Fig. 3:**
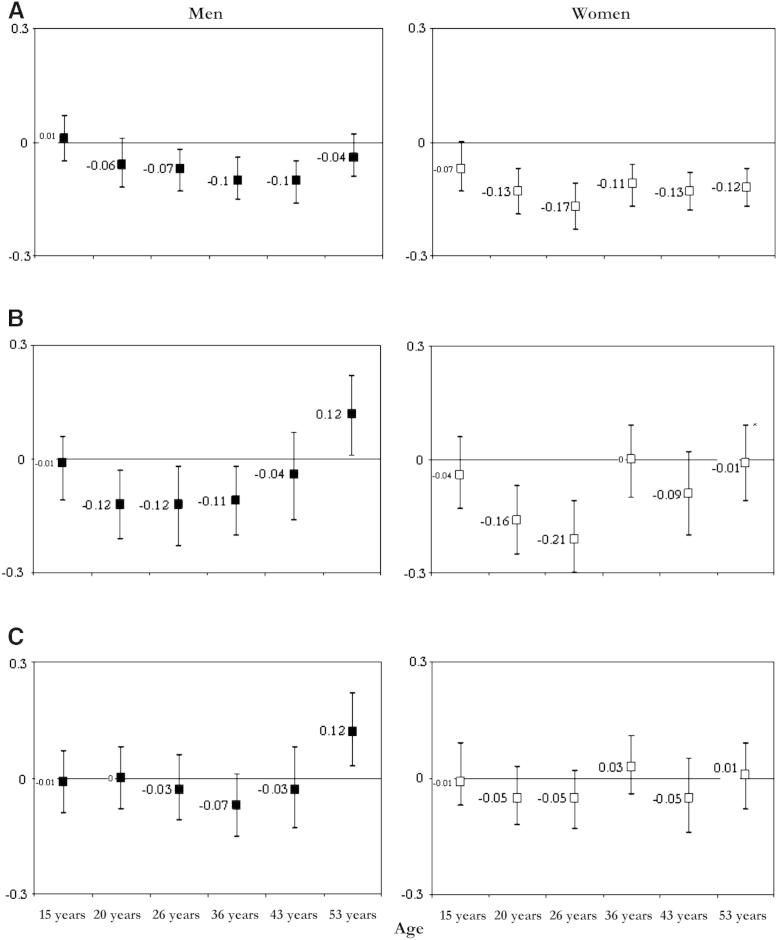
Regression coefficients (95% confidence interval [CI]) for the association between standardized cognitive scores of memory at age 53 years with mean *z* scores of BMI at different ages. Estimates are from unadjusted model, model 1 (adjusted for previous BMI), and model 3 (model 1 plus childhood cognition, education, and SEP). All analyses were carried out on the same sample with complete data on outcome, exposures, and covariates (see Supplementary Table 1). A: Unadjusted model; B: Model 1 (adjusted for previous BMI); C: Model 3 (model 1 plus childhood cognition, education, and SEP).

Each set of analysis was carried out on the sample of those with complete data for the relevant BMI and midlife cognitive measures and for all covariates added at stages to the unadjusted model (sample sizes are reported in detail in Supplementary Tables 1-3). In the unadjusted models, BMI was inversely associated with midlife memory, with few exceptions in men (BMI at age 15 years) and women (BMI at age 15 years and 20 years) (Fig. 3A). When previous BMI was adjusted (model 1), the magnitude of the inverse associations with memory slightly increased in both sexes for BMI at age 20 and 26 years, was attenuated at the other ages, and inverted at 53 years in men, when a direct cross-sectional association between BMI and memory (β per SD increase in BMI = 0.12; 95% confidence interval [CI]: 0.01, 0.22) emerged (Fig. 3; Supplementary Table 1). Associations were further attenuated when we adjusted for childhood cognitive ability (model 2), except for the positive association between high BMI and better memory at age 53 years in men (Supplementary Table 1). We found significant departures from linearity in women for BMI at age 26 years and 36 years in relation to memory at age 43 years (quadratic terms: β = −0.05; 95% CI: −0.09, −0.01 and β = −0.04; 95% CI: −0.08, −0.01, respectively) and for BMI from age 20 to 53 years in relation to memory at 53 years, suggesting that the unadjusted inverse associations between BMI and midlife memory function were curvilinear. To investigate the pattern of these relationships further, we entered BMI in the models as a categorical variable (according to World Health Organization criteria). Overall, progressively fewer participants fell into lower BMI categories with increasing age, such that by age 53 years, only four men and five women (0.3% of the total sample) were underweight (BMI: <18.5) as opposed to 1030 (28.7%) at age 15 years. Lower memory scores at 53 years were associated with both extremes of BMI at age 53 years in unadjusted models, such that compared with those having a normal weight, women who were underweight (β = −1.54; 95% CI: −2.52, −0.57) or obese (β = −0.30; 95% CI: −2.52, −0.57) had significantly worse memory, but the difference for the obese group associations was no longer significant when we adjusted for BMI at 43 years (*P* = .46).

**Fig. 4:**
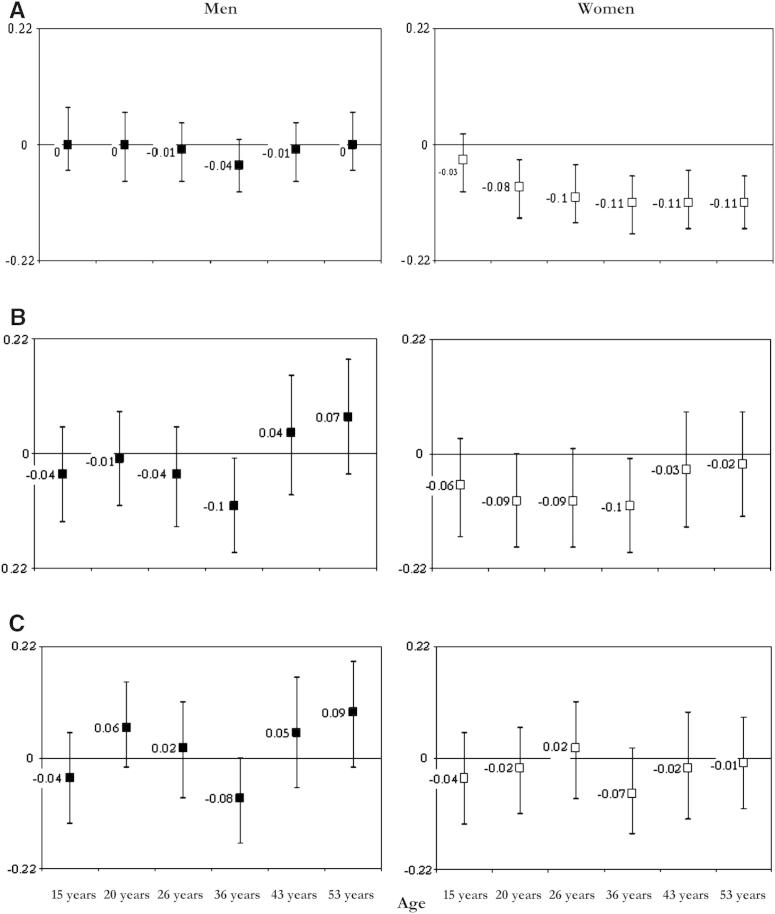
Regression coefficients (95% CI) for the association between standardized scores of verbal fluency at age 53 years with mean *z* scores of BMI at different ages. Estimates are from unadjusted model, model 1 (adjusted for previous BMI), and model 3 (model 1 plus childhood cognition, education, and SEP). All analyses are carried out on the same sample with complete data on outcome, exposures, and covariates (see Supplementary Table 1). A: Unadjusted; B: Model 1 (adjusted for previous BMI).

Unadjusted inverse associations between BMI and verbal fluency at age 53 years were statistically significant in women at all ages, except at 15 years, and in men at 36 years only (Fig. 4); gender interaction terms were significant (at α level: 0.10) at age 26 years (*P* = .066), 43 years (*P* = .080), and 53 years (*P* = .007). The magnitude of these associations in women was reduced when previous BMI (model 1) and childhood cognitive ability (model 2) were adjusted, with the single exception of BMI at age 36 years, which remained significant (Supplementary Table 1; Fig. 4). In men, the association between BMI at 36 years strengthened after adjustment for previous BMI and also remained significant after adjustment for childhood cognitive ability (Supplementary Table 1; Fig. 4); departures from linearity were statistically significant in women for BMI at age 20 years (*P* < .001), 26 years (*P* = .002), and 36 years (*P* = .006). Using BMI categories, we found only a nonsignificant trend toward lower verbal fluency scores among women who were underweight at age 20, 26, and 36 years compared with those having normal weight, whereas we found greater, statistically significant, lower verbal fluency scores among those overweight at age 20 years (β = −0.25; 95% CI: −0.49, −0.14) and 26 years (β = −0.22; 95% CI: −0.42, −0.27), but not at 36 years (*P* = .133), and among those obese at 20 years (β = −0.50; 95% CI: −0.99, −0.05), 26 years (β = −0.65; 95% CI: −1.08, −0.23), and 36 years (β = −0.40; 95% CI: −0.67, −0.13), compared with those of normal weight.

In both sexes, there were no associations between BMI at any age and the letter cancellation (speed) tests at age 43 (data not shown) and 53 years (Supplementary Table 1) in the unadjusted or adjusted models.

In the unadjusted models, only BMI in early midlife was associated with steeper 10-year decline in memory and attention (decline in letter search speed). All effects were weaker in men than in women, and were attenuated after adjusting for previous BMI (model 1) and childhood cognitive level (model 2), with the single exception found in women for BMI at 15 years and a 10-year decline in visual search accuracy (β = −0.08; 95% CI: −0.16, −0.00) (Supplementary Table 2).

### 3.3 BMI and midlife cognitive reserve

**Fig. 5:**
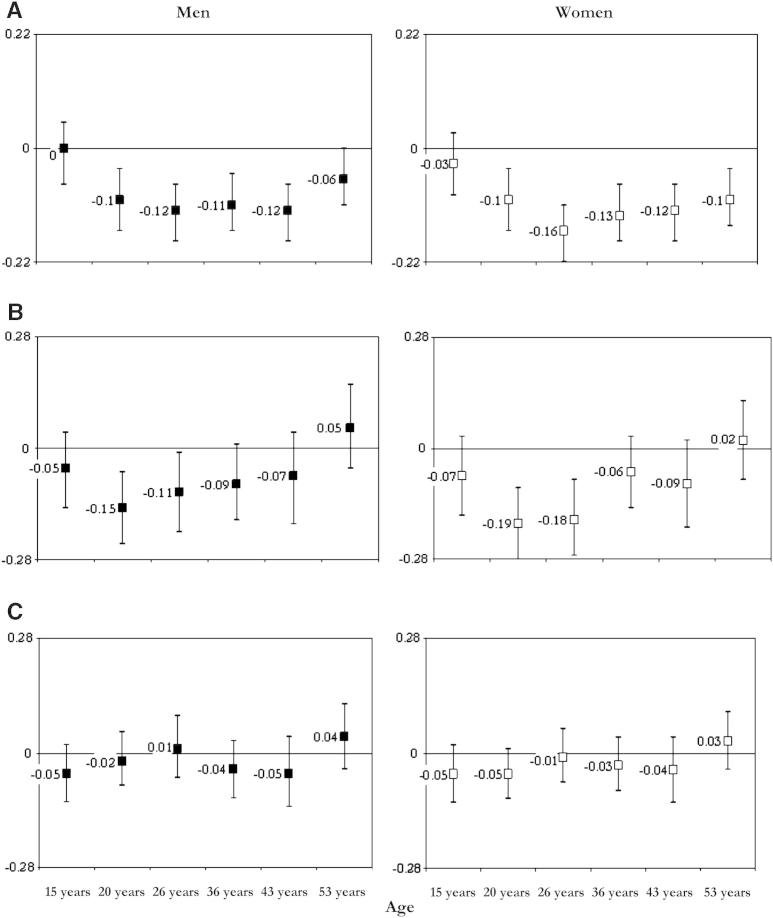
Regression coefficients (95% CI) for the association between standardized NART scores at age 53 years with mean *z* scores of BMI at different ages. Estimates are from unadjusted model, model 1 (adjusted for previous BMI), and model 3 (model 1 plus childhood cognition, education, and SEP). All analyses are carried out on the same sample with complete data on outcome, exposures, and covariates (see Supplementary Table 3). A: Unadjusted; B: Model 1 (adjusted for previous BMI); C: Model 3 (model 1 plus childhood cognition, education, and SEP).

For men and women, we found similar patterns of inverse associations for BMI at all ages and NART scores at age 53 years (Fig. 5); gender interaction terms were not significant at the 10% level. These associations remained significant at 20 and 26 years after adjusting for BMI at the previous age (model 1) in both men and women, suggesting that higher BMI gain at these ages is associated with lower cognitive reserve level in midlife (Supplementary Table 3; Fig. 5). When childhood cognitive ability was also adjusted (model 2), inverse associations remained statistically significant for BMI at 26 years and 36 years in women and for BMI at 36 years in men. Associations in women were nonlinear, and quadratic terms were significant for BMI at age 36 years (β = −0.04; 95% CI: −0.08, −0.01) and 53 years (β = −0.04; 95% CI: −0.08, −0.01). Further analyses using categorical BMI showed that at age 53 years, both underweight (β =−1.07; 95% CI: −1.95, −0.20) and obese (β = −0.27; 95% CI: −0.40, −0.14) women had significantly lower NART scores than women of normal weight. When we adjusted for previous BMI and childhood intelligence, only being underweight (β = −1.49; 95% CI: −2.51, −0.46) remained significantly associated with worse cognitive reserve. Conversely, there was no significant difference in NART scores between those of normal weight and those who were underweight at 36 years (*P* = .607) and 43 (*P* = .651) years, and those who were overweight at 43 years (*P* = .238) and 53 years (*P* = .714). Women who were overweight at 36 years and obese at 36, 43, and 53 years had lower NART scores than those of normal weight (all *P* values <.001).

In women only, there was an association between higher BMI gain at 36 years and lower enhancement of cognitive reserve, as measured by conditioning NART scores on verbal ability at age 26 years and adjusting for childhood intelligence (model 2) (β per SD increase in BMI =−0.06; 95% CI: −0.12, 0.00) (Supplementary Table 3).

### 3.4 Effect of education, SEP, lifestyle, and health characteristics

The association between BMI gain from 43 to 53 years and memory at 53 years was unchanged after additional adjustment for educational attainment and life course SEP (model 3). The magnitude of the associations between BMI change at 36 years and memory at 43 years and 53 years in men, and between BMI change at 26 years and memory at 53 years in women, was attenuated and no longer statistically significant (Supplementary Table 1; Fig. 3). We observed comparable attenuating effects of education and SEP on the associations between BMI and verbal fluency (Supplementary Table 1) and the NART, such that higher BMI gain at 36 years was no longer predictive of lower NART scores at 53 years (Supplementary Table 3). Only underweight women at age 53 years (β = −1.40; 95% CI: −2.35, −0.42) had independent significantly lower NART scores than women of normal weight.

In model 4, we additionally adjusted for physical activity level at age 43 and 53 years, self-reported mental and general health (General Health Questionnaire scores), diet (% of kilocalories from fats), and smoking habit, after which higher BMI change at 36 years was associated with lower memory scores at age 53 years in men (β per SD increase in BMI = −0.09; 95% CI: −0.18, −0.01), and higher BMI change at 53 years was still associated with better memory (β = 0.12; 95% CI: 0.01, 0.24); all associations between BMI and the NART (*P* > .295) and verbal fluency (*P* > .259) were nonsignificant. The effect of confounders included in model 4 was marked in women, and none of the associations between BMI at 26 years and memory at 53 years (*P* = .147), the NART (*P* = .228), and verbal fluency (*P* = .112) remained significant (data not shown).

Finally, when we additionally adjusted for blood pressure (at 43 and 53 years), cholesterol level, and glycated hemoglobin at age 53 years (model 5), the associations between BMI change at 36 years and memory at 53 years (*P* = .244) and the NART (*P* = .388) were no longer significant in men, but the direct association between high BMI gain and higher memory scores at age 53 years was unchanged in men (β = 0.12; 95% CI: 0.03, 0.24). The associations between high BMI gain at 36 years and lower NART scores (β = −0.09; 95% CI: −0.17, −0.01) became statistically significant in women, as did associations between high BMI gain at 36 years and lower verbal fluency in women (β = −0.11; 95% CI: −0.21, −0.02) and men (β = −0.12; 95% CI: −0.23, −0.01).

## 4 Discussion

In a representative British birth cohort study, BMI gain before midlife was associated with lower cognitive function in midlife independently of childhood cognitive function. However, these associations were largely explained by educational attainment and SEP. Conversely, BMI gain in midlife in men and women at age 53 years was associated with better memory at the same age, independent of aforementioned covariates, midlife vascular risk factors, and health and lifestyle characteristics.

There are limitations to our study. Loss to follow-up was more likely among those who were socially disadvantaged. However, the sample remained fairly representative of the national population of a similar age [16], and the modest differences in the distributions of the exposure (BMI) and the cognitive outcomes between those with complete and incomplete data suggest it would be unlikely that this would qualitatively alter our general findings. Next, at age 20 and 26 years, weight and height were self-reported. Although BMI measures at these ages may not be entirely consistent with those at other ages, it should be appreciated that height changes very little, if at all, up to mid-adulthood, and that self-reported weight is either fairly reliable or underestimated [27].

In general, previous studies on adiposity and cognitive function are based on samples of older adults (65+) and are extremely heterogeneous in their design and outcome measures [28]. Some were cross-sectional [29], [30], [31], had small sample sizes [32], [33], or had measured exposure (BMI/adiposity) only in older age [34], [35], [36]. Because our main findings refer to BMI gain rather than absolute BMI levels, comparisons with other studies are difficult to make. However, the absence of association between BMI gain and cognitive function in the present study after adjusting for relevant confounding factors, including educational level and SEP, is consistent with prospective studies reporting that overweight and obesity in midlife are not associated with verbal memory [36] and dementia [37] in old age. To our knowledge, there are only two other studies that have investigated the longitudinal effect of BMI from early midlife on cognitive function, the Whitehall II study in the United Kingdom [13] and the Vieillissement et Sante' au Travail (aging and health at work; VISAT) study in France [14]. Again, findings are not easily comparable, as results from the former were not stratified by gender, BMI at 25 years was self-reported several years later, and the study was based on absolute BMI rather than BMI change over time. Our study design is similar to and results are broadly consistent with the latter study. Although in the VISAT study childhood intelligence was not controlled for, there were no associations between BMI gain and cognitive performance when previous body size was adjusted and education and SEP were taken into account. These findings regarding the absence of an association between BMI gain in younger adulthood and memory scores in midlife are somewhat consistent with the similar lack of association between BMI early in life and dementia in old age found in the Prospective Population Study of Women in Sweden [11], but not with longitudinal evidence from the Kaiser Permanente medical care program in California, in which high BMI in early midlife conferred higher dementia risk [38]. In fact, in our study, men with higher BMI gain between ages 43 and 53 years had better memory, and both extremes of absolute BMI measures were associated with better memory function in women. These results must be interpreted with caution and deserve further investigation; however, they are, to some extent, consistent with the U-shaped association between BMI and lower cognitive function in late midlife reported in the Chicago Health and Aging Project (CHAP) study [35], and they seem to support the notion that lower cognitive function in midlife may lead to weight loss (and hence lower BMI), consistent with the previously reported association between Alzheimer’s disease and leptin, which promotes weight loss [39].

Because obesity is a component of the metabolic syndrome, our results seem inconsistent, with several studies suggesting that this syndrome is a risk factor for cognitive decline and dementia. However, these studies are typically based on older populations than NSHD, and also suggest that the metabolic syndrome is a risk factor for cognitive decline only in those with elevated inflammation [40].

There are different findings for the four cognitive tests we used. There was no association between high adiposity at any age and the letter cancellation test. This is consistent with findings on BMI and selective attention test scores in the VISAT study [14] but somewhat discordant with the Framingham Offspring [37] and the Maine–Syracuse Study [29] findings on a visuospatial test, namely, the B condition of the trail-making test [41]. However, while the test we applied is mainly sensitive to focal lesions and spatial neglect and is little affected by education [42], the trail B test requires efficient executive function, which decreases with age; therefore, it is possible that the relatively young age of participants explains the discrepancy. Memory measures are sensitive to cognitive decline since early midlife [43] and are the best discriminators of presymptomatic dementia [44]. The life course trend observed for BMI and memory was also observed for verbal fluency, ostensibly consistent with previous findings [45], although the associations between BMI gain at every age and this outcome were explained by the covariates. The same trend was also found for the NART, which, as a measure of cognitive “reserve” [46], might have been expected to augment in those with lower BMI gain during earlier adulthood, and has not previously been investigated in this context. Again, however, these associations were explained by the covariates.

## 5 Conclusion

The strengths of our study are the representativeness of our sample, which allows us to generalize our findings with confidence to similar populations, and the unique availability of measures of body size from early adulthood, along with valid and reliable tests of cognitive functions measured at two time points in midlife. Thus, the life course perspective offers an ideal approach to the association between adiposity and dementia [47]. From this strong vantage point, results from the 1946 British birth cohort study offer no evidence that weight gain up to early midlife is inversely and independently associated with subsequent cognitive function. However, they provide intriguing evidence that weight gain in later midlife may be protective in this respect. Further studies to elucidate potential mechanisms underlying this latter effect are clearly warranted.

## Figures and Tables

**Supplementary Table 1:** Regression coefficients (95% CI) for the association between cognitive function at age 53 years and unit increase in BMI at all ages

Body size	Sample size∗		Model 1†		Model 2‡		Model 3§	
Men	Women	Men	Women	Men	Women	Men	Women
Immediate-recall verbal memory at 43 years								
BMI 15	948	989	−0.06 (−0.16, 0.03)	−0.09 (−0.19, 0.01)	−0.06 (−0.15, 0.03)	−0.08 (−0.17, 0.01)	−0.04 (−0.12, 0.05)	−0.03 (−0.12, 0.06)
BMI 20	799	870	−0.10 (−0.19, −0.01)	−0.17 (−0.26, −0.07)	−0.03 (−0.12, 0.05)	−0.08 (−0.16, 0.01)	−0.01 (−0.09, 0.06)	−0.07 (−0.15, 0.01)
BMI 26	857	956	−0.12 (−0.22, −0.01)	−0.15 (−0.25, −0.05)	−0.06 (−0.15, 0.04)	−0.07 (−0.16, 0.02)	−0.02 (−0.10, 0.07)	−0.01 (−0.09, 0.08)
BMI 36	955	1042	−0.12 (−0.22, −0.03)	−0.04 (−0.13, 0.06)	−0.12 (−0.20, −0.03)	−0.05 (−0.14, 0.03)	−0.08 (−0.16, 0.01)	0.00 (−0.09, 0.08)
BMI 43	1046	1119	0.00 (−0.12, 0.12)	−0.10 (−0.21, 0.01)	0.03 (−0.08, 0.13)	−0.05 (−0.15, 0.05)	0.04 (−0.07, 0.14)	−0.06 (−0.15, 0.04)
Immediate-recall verbal memory at 53 years								
BMI 15	1037	1068	−0.01 (−0.11, 0.06)	−0.04 (−0.13, 0.06)	−0.02 (−0.11, 0.06)	−0.04 (−0.13, 0.04)	−0.01 (−0.09, 0.07)	−0.01 (−0.07, 0.09)
BMI 20	869	937	−0.12 (−0.21, −0.03)	−0.16 (−0.25, −0.07)	−0.03 (−0.11, 0.05)	−0.06 (−0.13, 0.02)	0.00 (−0.08, 0.08)	−0.05 (−0.12, 0.03)
BMI 26	918	1026	−0.12 (−0.23, −0.02)	−0.21 (−0.30, −0.11)	−0.06 (−0.15, 0.03)	−0.12 (−0.20, −0.04)	−0.03 (−0.11, 0.06)	−0.05 (−0.13, 0.02)
BMI 36	1014	1108	−0.11 (−0.20, −0.02)	0.00 (−0.10, 0.09)	−0.11 (−0.19, −0.02)	−0.02 (−0.10, 0.06)	−0.07 (−0.15, 0.01)	0.03 (−0.04, 0.11)
BMI 43	1173	1148	−0.04 (−0.16, 0.07)	−0.09 (−0.20, 0.02)	−0.02 (−0.12, 0.09)	−0.04 (−0.14, 0.05)	−0.03 (−0.13, 0.08)	−0.05 (−0.14, 0.05)
BMI 53	1151	1222	0.12 (0.01, 0.22)	−0.01 (−0.11, 0.09)	0.12 (0.03, 0.22)	−0.02 (−0.11, 0.07)	0.12 (0.03, 0.22)	0.01 (−0.08, 0.09)
Verbal fluency at 53 years								
BMI 15	1059	1087	−0.04 (−0.13, 0.05)	−0.06 (−0.16, 0.03)	−0.04 (−0.13, 0.04)	−0.07 (−0.16, 0.02)	−0.04 (−0.13, 0.05)	−0.04 (−0.13, 0.05)
BMI 20	885	949	−0.01 (−0.1, 0.08)	−0.09 (−0.18, 0.00)	0.05 (−0.03, 0.13)	−0.03 (−0.11, 0.06)	0.06 (−0.02, 0.15)	−0.02 (−0.11, 0.06)
BMI 26	930	1038	−0.04 (−0.14, 0.05)	−0.09 (−0.18, 0.01)	0.00 (−0.09, 0.10)	−0.03 (−0.12, 0.06)	0.02 (−0.08, 0.11)	0.02 (−0.08, 0.11)
BMI 36	1029	1127	−0.10 (−0.19, −0.01)	−0.10 (−0.19, −0.01)	−0.10 (−0.18, −0.01)	−0.11 (−0.20, −0.02)	−0.08 (−0.17, 0.00)	−0.07 (−0.15, 0.02)
BMI 43	1092	1168	0.04 (−0.08, 0.15)	−0.03 (−0.14, 0.08)	0.05 (−0.05, 0.16)	0.00 (−0.10, 0.10)	0.05 (−0.06, 0.16)	−0.02 (−0.12, 0.09)
BMI 53	1171	1242	0.07 (−0.04, 0.18)	−0.02 (−0.12, 0.08)	0.08 (−0.02, 0.18)	−0.04 (−0.13, 0.06)	0.09 (−0.02, 0.19)	−0.01 (−0.1, 0.08)
Letter cancellation speed at 53 years								
BMI 15	942	992	−0.03 (−0.13, 0.07)	−0.02 (−0.12, 0.08)	−0.03 (−0.13, 0.06)	−0.03 (−0.12, 0.07)	−0.02 (−0.12, 0.07)	−0.01 (−0.11, 0.08)
BMI 20	853	915	−0.03 (−0.11, 0.06)	−0.01 (−0.11, 0.08)	0.00 (−0.09, 0.09)	0.01 (−0.08, 0.1)	0.01 (−0.08, 0.1)	0.01 (−0.08, 0.11)
BMI 26	911	1008	−0.07 (−0.17, 0.03)	−0.04 (−0.14, 0.06)	−0.04 (−0.15, 0.06)	−0.02 (−0.11, 0.08)	−0.03 (−0.13, 0.07)	−0.01 (−0.11, 0.09)
BMI 36	1001	1094	−0.05 (−0.14, 0.05)	−0.02 (−0.11, 0.08)	−0.04 (−0.14, 0.05)	−0.02 (−0.11, 0.07)	−0.02 (−0.12, 0.07)	−0.02 (−0.11, 0.08)
BMI 43	1055	1130	−0.09 (−0.21, 0.03)	−0.11 (−0.22, 0.00)	−0.08 (−0.2, 0.04)	−0.09 (−0.2, 0.02)	−0.08 (−0.2, 0.04)	−0.09 (−0.2, 0.02)
BMI 53	1129	1195	−0.02 (−0.13, 0.09)	0.02 (−0.08, 0.12)	−0.01 (−0.12, 0.09)	0.02 (−0.08, 0.12)	−0.03 (−0.13, 0.08)	0.02 (−0.08, 0.12)

Abbreviations: BMI, body mass index; SEP, socioeconomic position; 95% CI, 95% confidence interval.

NOTE. Positive coefficients represent better cognitive function.

∗ Analyses are carried out on the same sample with complete data on all relevant variables at each follow-up.

† Adjusted for BMI at previous follow-up (BMI at age 15 years is adjusted for BMI at age 11 years).

‡ Model 1 plus childhood cognitive ability at age 8 years.

§ Model 2 plus education attainment and lifelong SEP.

**Supplementary Table 2:** Regression coefficients (95% CI) representing rate of cognitive decline per SD increase of BMI

	Sample size∗		Model 1†		Model 2‡		Model 3§	
Men	Women	Men	Women	Men	Women	Men	Women
10-year memory decline								
BMI 15	937	973	0.01 (−0.05, 0.07)	−0.03 (−0.08, 0.03)	−0.01 (−0.06, 0.05)	−0.04 (−0.09, 0.01)	−0.02 (−0.07, 0.03)	−0.05 (−0.10, 0.00)
BMI 20	791	858	−0.04 (−0.11, 0.03)	−0.07 (−0.14, 0.00)	−0.01 (−0.08, 0.06)	−0.04 (−0.11, 0.03)	0.01 (−0.07, 0.08)	−0.04 (−0.11, 0.03)
BMI 26	850	941	−0.05 (−0.13, 0.03)	−0.08 (−0.15, 0.00)	−0.03 (−0.11, 0.04)	−0.05 (−0.12, 0.02)	−0.02 (−0.10, 0.06)	−0.02 (−0.10, 0.05)
BMI 36	944	1023	−0.04 (−0.11, 0.04)	0.03 (−0.05, 0.10)	−0.04 (−0.11, 0.03)	0.02 (−0.05, 0.08)	−0.02 (−0.10, 0.05)	0.04 (−0.03, 0.11)
BMI 43	1033	1099	−0.06 (−0.15, 0.04)	−0.03 (−0.11, 0.05)	−0.04 (−0.13, 0.05)	−0.01 (−0.09, 0.07)	−0.06 (−0.15, 0.03)	−0.01 (−0.09, 0.07)
10-year visual search decline								
BMI 15	877	923	−0.02 (−0.1, 0.07)	−0.01 (−0.1, 0.08)	−0.01 (0.73, −0.1)	−0.01 (0.77, −0.1)	−0.01 (0.76, −0.1)	−0.01 (0.8, −0.1)
BMI 20	797	852	−0.02 (−0.1, 0.05)	−0.01 (−0.1, 0.07)	−0.01 (0.82, −0.08)	0.00 (0.95, −0.08)	−0.01 (0.88, −0.08)	0.00 (0.95, −0.08)
BMI 26	853	937	−0.06 (−0.14, 0.03)	0.00 (−0.09, 0.08)	−0.04 (0.33, −0.13)	0.01 (0.79, −0.08)	−0.04 (0.39, −0.13)	0.01 (0.8, −0.08)
BMI 36	946	1022	0.00 (−0.08, 0.08)	0.01 (−0.07, 0.09)	0.00 (0.95, −0.08)	0.00 (0.91, −0.08)	0.00 (0.94, −0.08)	0.00 (0.93, −0.08)
BMI 43	1031	1095	−0.05 (−0.16, 0.05)	−0.05 (−0.14, 0.05)	−0.05 (0.32, −0.15)	−0.04 (0.44, −0.13)	−0.05 (0.29, −0.16)	−0.04 (0.43, −0.13)

NOTE. Positive coefficients indicate slower decline.

∗ Analyses are carried out on the same sample with complete data on all relevant variables at each follow-up.

† Adjusted for earlier BMI as appropriate.

‡ Model 1 plus childhood cognitive ability at age 8 years.

§ Model 2 plus education attainment and lifelong SEP.

**Supplementary Table 3:** Regression coefficients (95% CI) for the associations between unit increase in BMI and NART scores at 53 years, and NART scores at 53 years conditioned to verbal ability scores obtained at age 26 years

	Sample size∗		Model 1†		Model 2‡		Model 3§	
Men	Women	Men	Women	Men	Women	Men	Women
NART score at 53 years								
BMI 15	1015	1050	−0.05 (−0.15, 0.04)	−0.07 (−0.17, 0.03)	−0.07 (−0.14, 0.01)	−0.08 (−0.16, 0.01)	−0.05 (−0.12, 0.02)	−0.05 (−0.12, 0.02)
BMI 20	851	920	−0.15 (−0.24, −0.06)	−0.19 (−0.28, −0.1)	−0.03 (−0.1, 0.04)	−0.06 (−0.13, 0.01)	−0.02 (−0.08, 0.05)	−0.05 (−0.11, 0.01)
BMI 26	897	1007	−0.11 (−0.21, −0.01)	−0.18 (−0.27, −0.08)	−0.03 (−0.1, 0.05)	−0.07 (−0.14, 0.00)	0.01 (−0.06, 0.09)	−0.01 (−0.07, 0.06)
BMI 36	983	1087	−0.09 (−0.18, 0.01)	−0.06 (−0.15, 0.03)	−0.08 (−0.15, 0.00)	−0.08 (−0.15, −0.01)	−0.04 (−0.11, 0.03)	−0.03 (−0.09, 0.04)
BMI 43	1046	1125	−0.07 (−0.19, 0.04)	−0.09 (−0.2, 0.02)	−0.04 (−0.13, 0.05)	−0.04 (−0.12, 0.05)	−0.05 (−0.13, 0.04)	−0.04 (−0.12, 0.04)
BMI 53	1121	1199	0.05 (−0.05, 0.16)	0.02 (−0.08, 0.12)	0.07 (−0.02, 0.15)	0.00 (−0.07, 0.08)	0.04 (−0.04, 0.12)	0.03 (−0.04, 0.10)
27-year enhancement in NART score¶								
BMI 15	924	976	−0.02 (−0.07, 0.03)	0.00 (−0.05, 0.05)	−0.03 (−0.08, 0.01)	−0.02 (−0.06, 0.03)	−0.04 (−0.08, 0.01)	−0.02 (−0.07, 0.03)
BMI 20	787	866	−0.05 (−0.12, 0.01)	−0.05 (−0.11, 0.01)	−0.03 (−0.09, 0.03)	−0.03 (−0.09, 0.03)	−0.03 (−0.09, 0.03)	−0.03 (−0.09, 0.03)
BMI 26	897	1006	0.01 (−0.07, 0.08)	−0.07 (−0.13, 0.00)	0.01 (−0.05, 0.08)	−0.05 (−0.11, 0.01)	0.03 (−0.04, 0.09)	−0.02 (−0.08, 0.04)
BMI 36	983	1086	0.00 (−0.07, 0.07)	−0.04 (−0.1, 0.02)	−0.02 (−0.08, 0.04)	−0.06 (−0.12, 0.00)	−0.01 (−0.07, 0.05)	−0.03 (−0.09, 0.03)
BMI 43	979	1067	−0.05 (−0.14, 0.04)	−0.03 (−0.11, 0.05)	−0.03 (−0.12, 0.05)	−0.01 (−0.09, 0.06)	−0.04 (−0.12, 0.04)	−0.01 (−0.09, 0.06)

Abbreviation: NART, National Adult Reading Test.

NOTE. Negative coefficients represent lower level “cognitive reserve” level at age 53 years or of enhancement between 26 and 53 years.

∗ Analyses are carried out on the same sample with complete data on all relevant variables at each follow-up.

† Adjusted for earlier BMI as appropriate.

‡ Model 1 plus childhood cognitive ability at age 8 years.

§ Model 2 plus education attainment and lifelong SEP.

¶ These models are conditioned to verbal ability scores at age 26 years measured by means of the Watts–Vernon Reading Test.

**Table 1:** Stages of covariate adjustment (ages at measurement)

Model 1	Model 2	Model 3	Model 4	Model 5
Previous BMI	Previous BMI	Previous BMI	Previous BMI	Previous BMI
	Childhood IQ (8 years)	Childhood IQ (8 years)	Childhood IQ (8 years)	Childhood IQ (8 years)
		Education (26 years)	Education (26 years)	Education (26 years)
		SEP (15–53 years)	SEP (15–53 years)	SEP (15–53 years)
			Smoking (20–53 years)	Smoking (20–53 years)
			Exercise (43, 53HbA1)	Exercise (43, 53 years)
			GHQ (53 years)	GHQ (53 years)
			Diet∗ (36, 43, 53 years)	Diet (36, 43, 53 years)
				BP (43, 53 years)
				Cholesterol (53 years)
				HbA1C (53 years)

Abbreviations: BMI, body mass index; SEP, socioeconomic position; BP, blood pressure; GHQ, General Health Questionnaire; HbA1C, glycated hemoglobin (%).

∗ Percent of kilocalories from fats.

**Table 2:** Characteristics of participants with complete data for all cognitive measures, BMI measures at 43 and 53 years, and all covariates through model 3 (education and SEP) (n∗ = 2083)

	Men	Women
Education (at 26 years), n (%)	n = 1013	n = 1070
No qualification	321 (31.7)	368 (34.4)
Vocational	64 (6.3)	102 (9.5)
Ordinary secondary	159 (15.7)	283 (26.5)
Advanced secondary	310 (30.6)	259 (24.2)
Higher	159 (15.7)	58 (5.4)
SEP (15–53 years), n (%)	n = 1013	n = 1070
Professional	125 (12.3)	16 (1.5)
Intermediate	428 (42.3)	366 (34.2)
Nonmanual skilled	91 (9)	404 (37.8)
Manual skilled	282 (27.8)	72 (6.7)
Partly skilled	71 (7)	169 (15.8)
Unskilled	16 (1.6)	43 (4)
Smoking (20–53 years), n (%)	n = 989	n = 1047
Never smoker	253 (25.6)	320 (30.6)
Predominantly nonsmoker	371 (37.5)	358 (34.2)
Predominantly smoker	225 (22.8)	212 (20.3)
Lifelong smoker	140 (14.2)	157 (15)
Exercise (43 years), n (%)	n = 1013	n = 1070
None	460 (45.4)	585 (54.7)
1–4 times a month	161 (15.9)	152 (14.2)
4+ times a month	392 (38.7)	333 (31.1)
Exercise (53 years), n (%)	n = 1013	n = 1070
None	451 (44.5)	522 (48.8)
1–4 times a month	215 (21.2)	184 (17.2)
4+ times a month	346 (34.2)	364 (34)
GHQ (53 years), mean (SD) (n)	17.9 (4.5) (n = 1004)	19.3 (5.4) (n = 1057)
% of kilocalories from fats (36, 43, 53 years), mean (SD) (n)	41.5 (5.1) (n = 902)	42.3 (5.4) (n = 957)
Blood pressure (mm/Hg), mean (SD)	n = 1004	n = 1050
Diastolic (43 years)	81.4 (11.4)	76.5 (11.4)
Systolic (43 years)	124.5 (15.1)	121 (15.6)
Diastolic (53 years)	87.1 (11.8)	82.2 (11.7)
Systolic (53 years)	139.6 (19.5)	132.8 (19.4)
Cholesterol (mmol/L) (53 years), mean (SD) (n)	6 (1.1) (n = 891)	6.1 (1.1) (n = 920)
HbA1c (% of total serum hemoglobin) (53 years), mean (SD) (n)	5.6 (0.7) (n = 897)	5.6 (0.6) (n = 920)

∗ Smaller sample sizes are reported as appropriate for covariates included in models 4 and 5.

## References

[1] Prince M., Jackson J.C., Albanese E., Sousa R.M., Ferri C.P., Ribeiro W.S. (2009). World Alzheimer report. Alzheimer’s Disease International.

[2] Skoog I., Kalaria R.N., Breteler M.M. (1999). Vascular factors and Alzheimer disease. Alzheimer Dis Assoc Disord.

[3] Yaffe K. (2007). Metabolic syndrome and cognitive decline. Curr Alzheimer Res.

[4] Luchsinger J.A., Mayeux R. (2004). Dietary factors and Alzheimer’s disease. Lancet Neurol.

[5] Ogden C.L., Carroll M.D., Curtin L.R., McDowell M.A., Tabak C.J., Flegal K.M. (2006). Prevalence of overweight and obesity in the United States, 1999-2004. JAMA.

[6] Gustafson D. (2008). A life course of adiposity and dementia. Eur J Pharmacol.

[7] Gorospe E.C., Dave J.K. (2007). The risk of dementia with increased body mass index. Age Ageing.

[8] Barrett Connor E., Edelstein S.L., Corey-Bloom J., Wiederholt W.C. (1996). Weight loss preceded dementia in community dwelling older adults. J Am Geriatr Soc.

[9] Knopman D.S., Edland S.D., Cha R.H., Petersen R.C., Rocca W.A. (2007). Incident dementia in women is preceded by weight loss by at least a decade. Neurology.

[10] Grundman M., Corey-Bloom J., Jernigan T., Archibald S., Thal L.J. (1996). Low body weight in Alzheimer’s disease is associated with mesial temporal cortex atrophy. Neurology.

[11] Gustafson D.R., Backman K., Waern M., Ostling S., Guo X., Zandi P., Mielke M.M., Bengtsson C., Skoog I. (2009). Adiposity indicators and dementia over 32 years in Sweden. Neurology.

[12] Whitmer R.A., Gustafson D.R., Barrett-Connor E., Haan M.N., Gunderson E.P., Yaffe K. (2008). Central obesity and increased risk of dementia more than three decades later. Neurology.

[13] Sabia S., Kivimaki M., Shipley M.J., Marmot M.G., Singh-Manoux A. (2009). Body mass index over the adult life course and cognition in late midlife: the Whitehall II Cohort Study. Am J Clin Nutr.

[14] Cournot M., Marquie J.C., Ansiau D., Martinaud C., Fonds H., Ferrières J., Ruidavets J.B. (2006). Relation between body mass index and cognitive function in healthy middle-aged men and women. Neurology.

[15] Wadsworth M., Kuh D., Richards M., Hardy R. (2006). Cohort profile: The 1946 national birth cohort (MRC National Survey of Health and Development). Int J Epidemiol.

[16] Wadsworth M.E., Butterworth S.L., Hardy R.J., Kuh D.J., Richards M., Langenberg C., Hilder W.S., Connor M. (2003). The life course prospective design: an example of benefits and problems associated with study longevity. Soc Sci Med.

[17] Nelson H.E., Willison J.R. (1991). National adult reading test (NART).

[18] Richards M., Hardy R., Wadsworth M.E. (2005). Alcohol consumption and midlife cognitive change in the British 1946 birth cohort study. Alcohol Alcohol.

[19] Douglas J.W.B., Ross J.M., Simpson H.R., Pigeon D.A. (1968). Details of the fifteen years tests.

[20] Plassman B.L., Williams J.W., Burke J.R., Holsinger T., Benjamin S. (2010). Systematic review: factors associated with risk for and possible prevention of cognitive decline in later life. Ann Intern Med.

[21] Kivipelto M., Ngandu T., Fratiglioni L., Viitanen M., Kåreholt I., Winblad B. (2005). Obesity and vascular risk factors at midlife and the risk of dementia and Alzheimer disease. Arch Neurol.

[22] Surveys OoPCa (1970). Classification of occupations.

[23] Goldberg D.P., Hillier V.F. (1979). A scaled version of the General Health Questionnaire. Psychol Med.

[24] Prynne C.J., Paul A.A., Mishra G.D., Greenberg D.C., Wadsworth M.E. (2005). Changes in intake of key nutrients over 17 years during adult life of a British birth cohort. Br J Nutr.

[25] Langenberg C., Kuh D., Wadsworth M.E., Brunner E., Hardy R. (2006). Social circumstances and education: life course origins of social inequalities in metabolic risk in a prospective national birth cohort. Am J Public Health.

[26] STATA (2007). Stata statistical software: release 10.

[27] Nyholm M., Gullberg B., Merlo J., Lundqvist-Persson C., Råstam L., Lindblad U. (2007). The validity of obesity based on self-reported weight and height: implications for population studies. Obesity (Silver Spring).

[28] Gustafson D. (2006). Adiposity indices and dementia. Lancet Neurol.

[29] Dore G.A., Elias M.F., Robbins M.A., Budge M.M., Elias P.K. (2008). Relation between central adiposity and cognitive function in the Maine-Syracuse Study: attenuation by physical activity. Ann Behav Med.

[30] Jagust W., Harvey D., Mungas D., Haan M. (2005). Central obesity and the aging brain. Arch Neurol.

[31] Nourhashemi F., Andrieu S., Gillette-Guyonnet S., Reynish E., Albarède J.L., Grandjean H., Vellas B. (2002). Is there a relationship between fat-free soft tissue mass and low cognitive function? Results from a study of 7,105 women. J Am Geriatr Soc.

[32] Gunstad J., Paul R.H., Cohen R.A., Tate D.F., Spitznagel M.B., Grieve S., Gordon E. (2008). Relationship between body mass index and brain volume in healthy adults. Int J Neurosci.

[33] Sweat V., Starr V., Bruehl H., Arentoft A., Tirsi A., Javier E., Convit A. (2008). C-reactive protein is linked to lower cognitive performance in overweight and obese women. Inflammation.

[34] Kanaya A.M., Barrett-Connor E., Gildengorin G., Yaffe K. (2004). Change in cognitive function by glucose tolerance status in older adults: a 4-year prospective study of the Rancho Bernardo study cohort. Arch Intern Med.

[35] Sturman M.T., de Leon C.F., Bienias J.L., Morris M.C., Wilson R.S., Evans D.A. (2008). Body mass index and cognitive decline in a biracial community population. Neurology.

[36] West N.A., Haan M.N. (2009). Body adiposity in late life and risk of dementia or cognitive impairment in a longitudinal community-based study. J Gerontol A Biol Sci Med Sci.

[37] Wolf P.A., Beiser A., Elias M.F., Au R., Vasan R.S., Seshadri S. (2007). Relation of obesity to cognitive function: importance of central obesity and synergistic influence of concomitant hypertension. The Framingham Heart Study. Curr Alzheimer Res.

[38] Whitmer R.A., Gunderson E.P., Barrett-Connor E., Quesenberry C.P., Yaffe K. (2005). Obesity in middle age and future risk of dementia: a 27 year longitudinal population based study. BMJ.

[39] Lieb W., Beiser A.S., Vasan R.S., Tan Z.S., Au R., Harris T.B. (2009). Association of plasma leptin levels with incident Alzheimer disease and MRI measures of brain aging. JAMA.

[40] Yaffe K., Haan M., Blackwell T., Cherkasova E., Whitmer R.A., West N. (2007). Metabolic syndrome and cognitive decline in elderly Latinos: findings from the Sacramento Area Latino Study of Aging study. J Am Geriatr Soc.

[41] Reitan R.M., Wolfson D. (1993). The Halstead-Reitan Neuropsychological Test battery: theory and clinical interpretation.

[42] Uttl B., Pilkenton-Taylor C. (2001). Letter cancellation performance across the adult life span. Clin Neuropsychol.

[43] Salthouse T. (2003). Memory aging from 18 to 80. Alzheimer Dis Assoc Disord.

[44] Chen P., Ratcliff G., Belle S.H., Cauley J.A., DeKosky S.T., Ganguli M. (2007). Cognitive tests that best discriminate between presymptomatic AD and those who remain nondemented. Neurology.

[45] Brubacher D., Monsch A.U., Stahelin H.B. (2004). Weight change and cognitive performance. Int J Obes Relat Metab Disord.

[46] Richards M., Deary I.J. (2005). A life course approach to cognitive reserve: a model for cognitive aging and development?. Ann Neurol.

[47] Ben-Shlomo Y., Kuh D. (2002). A life course approach to chronic disease epidemiology: conceptual models, empirical challenges and interdisciplinary perspectives. Int J Epidemiol.

